# Prenatal Diagnosis of Complete Atrioventricular Septal Defect: Perinatal and Neonatal Outcomes

**DOI:** 10.1155/2009/958496

**Published:** 2009-06-04

**Authors:** Gokhan Yıldırım, Kemal Gungorduk, Fehmi Yazıcıoğlu, Ahmet Gul, Fatma Çakar, Özgü Çelikkol, Yavuz Ceylan

**Affiliations:** ^1^Maternal and Fetal Unit, Department of Obstetrics and Gynecology, Istanbul Bakirkoy Women and Children Hospital, Istanbul, Turkey; ^2^Atakent Mah. Soyak Olimpiaket Sitesi, D-13/57, 34303 Istanbul, Turkey; ^3^Maternal and Fetal Unit, Department of Obstetrics and Gynecology, Istanbul Sulaymanıye Women and Children Hospital, Istanbul, Turkey

## Abstract

*Objective*. The purpose of this study was to establish the outlook for fetuses diagnosed with complete atrioventricular septal defect (cAVSD) prenatally and its relation to additional cardiac, extracardiac, and chromosomal abnormalities. *Methods*. We retrospectively reviewed fetal echocardiograms diagnosed with cAVSD from January 2002 to December 2007, comparing fetuses with and without aneuploidy. *Results*. Complete antrioventricular septal defect was confirmed in 62 fetuses. Mean maternal age was 28.79 ± 4.78 years (range 20–38). Mean gestational age was 23.69 ± 5.48 weeks (range 12–38). Fetal karyotype was known in all fetuses. An abnormal karyotype was found in 21 fetuses. Complete AVSD occurred without any other intracardiac abnormality in 28 fetuses. Extracardiac anomalies were present in 38 fetuses. As for pregnancy outcomes, there were 36 (58%) terminations of pregnancy and 4 (6.4%) intrauterine fetal deaths. In these four fetuses, complex cAVSD was associated with atrioventricular block (one case), heterotaxy (one case), and fetal hydrops (two cases). Of the 22 live births, 5 were neonatal deaths without surgery while 17 babies underwent surgery and 13 have survived to date. The mean survival age was 53 ± 4 months (range 22–64 m). *Conclusion*. AVSD is associated with chromosomal, other cardiac, and extracardiac abnormalities. The detection of these abnormalities is important in order to give the best indication of the likely outcome when counselling parents.

## 1. Introduction

Atrioventricular septal defects (AVSDs) also known as endocardial cushion defects involve the septal portions of the mitral and tricuspid valves and the adjacent atrial and ventricular septum [1]. It is a common cardiac defect in prenatal life [2]. The estimated incidence of the condition varies from 0.33/1000 live births to 0.51/1000 live births [3]. Complete AVSD (cAVSD) is identified by the echocardiographic hallmark of a common atrioventricular valve and the distortion of the normal appearance at the crux of the heart.

Prenatal detection of cAVSD is very important because it is usually associated with chromosomal abnormalities such as Trisomy 21, which occurs in at least 50% of all cases [4]. It is often associated with other cardiac and extracardiac anomalies [1, 2].

The purpose of this retrospective study was conducted to estimate the frequency of other cardiac, extracardiac, and chromosomal anomalies and neonatal outcomes in fetuses with cAVSD diagnosed in our maternal perinatal units.

## 2. Methods

**Figure 1:**
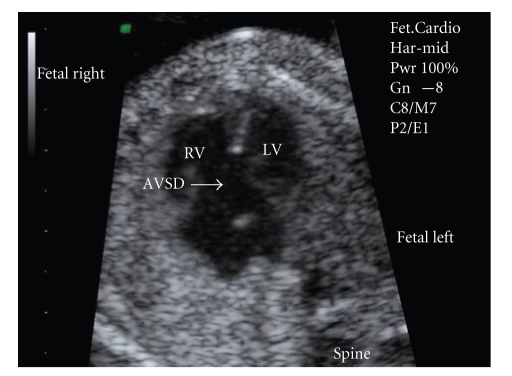
Apical four-chamber view showing a defect of isolated complete atrioventricular septal defet. AVSD: atrioventricular septal defet; RV: right ventricle; LV: left ventricle.

This was a retrospective study conducted between January 2002 and December 2007 at the Department of Maternal Fetal Medicine of the Bakirkoy Women and Children's Teaching Hospital and the Department of Maternal Fetal Medicine of the Sulaymaniye Women's Teaching Hospital. These hospitals serve as a tertiary referral center in Istanbul. We reviewed all 62 cases of complete atrioventricular septal defects diagnosed prenatally. Ultrasound examinations were carried out using a Voluson 730 Expert (GE Healthcare, Chalfont St Giles, UK). All patients underwent two dimensional, color and pulsed Doppler echocardiography. M-mode tracing was used in case of arrhythmias and whenever necessary. The diagnosis of an atrioventricular septal defect relies on the following sonographic markers: (1) atrioventricular valves inserting into a common junction or in a linear fashion instead of the normal differential insertion; (2) a large defect “discontinuity” located in the cushion zone of the atrioventricular septum; (3) atrial and ventricular septal defects of varying size; (4) aorta “unsprung” from the crux of the heart; (5) unstable floating “kicking” crux (Figure 1).

Prenatal karyotyping was offered to all parents and performed in all cases. The mothers choosing to continue the pregnancy were followed up and were examined usually twice or three times after diagnosis. The neonates were studied after birth. If the parents opted for termination of the pregnancy, postmortem examination of the fetus was advised. All parents agreed to proceed a postmortem examination. The vaginal misoprostol dose for pregnancy termination is usually 200 *μ*g every 6 hours.

Details of the cardiac anatomy were determined from prenatal and postnatal echocardiograms or other postnatal investigations, from surgical findings or at autopsy. Extracardiac abnormalities and syndromes that were detected prenatally or only became apparent postnatally were noted.

The relative size of the ventricles was classified as approximately equal in size, right ventricle substantially larger than the left (dominant right/Hypoplasia of the left heart) or left ventricle substantially larger than the right (dominant left/Hypoplasia of the right heart). Left isomerism was diagnosed in the presence of azygos continuation of an interrupted inferior vena cava and/or fetal heart block, supported by structural heart disease and viscerocardiac heterotaxy (stomach visualized contralateral to the cardiac apex). A diagnosis of right isomerism was made in the presence of a combination of complete atrioventricular septal defect and/or viscerocardiac heterotaxy and/or juxtaposition of inferior vena cava and aorta on the same side of the spine.

The cases were analyzed for associated cardiac and noncardiac anomalies, karyotyp7e, and obstetric and neonatal outcomes. Isolated cAVSD was defined as an isolated lesion at the fetal echocardiogram. A diagnosis of complex CAVSD was only considered when additional intracardiac malformation was seen at the fetal echocardiogram. Demographic data including maternal age, parity, gestational age at diagnosis, and reason for referral were also noted. 

MedCalc 9.3 program was used for statistical analysis. Normal distribution of continuous variables was assessed by Kolmogrov-Smimov test. Chi-square analysis was used for analysis of categoric variables, Student-*t* test was used for normal distributed variables in the analysis of continuous variables and Mann Whitney *U* test was used for abnormally distributed variables. Relative risk (RR) with 95% confidence interval (CI) was calculated. A *P* value of less than .05 was considered statistically significant.

## 3. Results

We reviewed all 62 cases of complete atrioventricular septal defect diagnosed prenatally. In all cases the diagnosis was confirmed by postmortem examination or postnatal examination. Demographic data are presented in Table 1. All were singleton pregnancies. Indications for performing fetal echocardiograpy during the study period are shown in Table 2. Abnormal four-chamber view was the primary indication for referral in 16 fetuses (25%). The atrioventricular septal defect was an isolated cardiac lesion at the fetal echocardiogram in 28 (45.1%) cases. All of the other fetuses had additional intracardiac malformations (e.g., left ventricular hypoplasia, double-outlet right ventricle, and left or right isomerism). Detailed data are shown in Table 3. Of 12 fetuses diagnosed with heterotaxy syndrome prenatally, 7 had right atrial isomerism and 5 had left atrial isomerism.

Associated extracardiac anomalies were present in 38 cases (63.3%). There was a single extracardiac anomaly in 12 fetuses, two anomalies in 10 cases and >2 in 16 cases. Associated extracardiac anomalies by apparatus are shown in Table 4. The demographic differences and outcomes between isolated and complex cAVSD are shown in Table 5. There was no statistically significant difference in the frequency of termination in the presence of isolated cardiac anatomy (53%) compared with those with more complex anatomy (61%) [*P* =.60; RR: 0.71; 95% CI: 0.25–1.97]. Also there was not any statistical significance in the frequency of chromosomal anomalies between the two groups [*P* = 1.0; RR: 0.86; 95% CI: 0.30–2.50]. 

**Figure 2:**
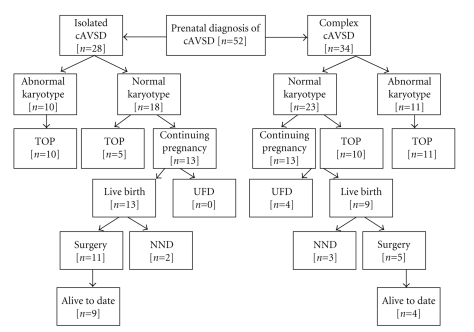
Flow diagram of the outcome of prenatally diagnosed fetuses with complete AVSD. TOP: elective termination of pregnancy; IUDF: Intrauterine fetal death; NND: neonatal death (28 days).

Fetal karyotype was known in all fetuses. Chromosomal anomalies were detected in 21 fetuses (33%): ten had trisomy 21, seven had trisomy 18, three had trisomy 13, and one had triploidy. As for pregnancy outcomes, there were 36 (58%) terminations of pregnancy and 4 (6.4%) intrauterine fetal deaths. Detailed information about these four cases are summarized in Table 6. Of the 22 live births, 5 were neonatal deaths without surgery while 17 babies underwent surgery and 13 have survived to date. The mean survival age was 53 ± 4 months (range 22–64 m). The outcomes of fetuses are summarized in Figure 2.

## 4. Discussion

Atrioventricular septal defects (AVSDs) are characterized by defects in the atrial and ventricular septum immediately above and below the atrioventricular (AV) valves (tricuspid and mitral). Traditionally, this congenital abnormality has also been referred to as AV canal defect, endocardial cushion defect, persistent atrioventricular ostium, and canalis atrioventricularis communis. The defects usually involve the AV valves to some degree, and the pathophysiology of the lesion depends on the extent of shunting at both the atrial and ventricular levels as well as regurgitation from the involved valves. When limited to the atrial septum, the condition is called an ostium primum atrial septal defect (primum ASD) or partial AVSD. Complete AVSDs combine deficiency of both the atrial and ventricular septum with severe abnormality of the mitral and tricuspid valves, creating what is in essence a common AV valve that serves both ventricles [5].

An AVSD is a common cardiac condition detected prenatally, accounting for 17 percent of diagnosis in the Allan and Sharland series and approximately 4 to 5 percent of congenital heart disease in live births [4, 6].

Antenatal diagnosis of complete atrioventricular septal defects is not always easy. When the atrial and septal defects are large, the four-chamber view reveals an obvious deficiency of the central core structures of the heart. Color Doppler ultrasound can be useful in that it facilitates the visualization of the central opening of the single atrioventricular valve. The atria may be dilated as a consequence of atrioventricular insufficiency. In such cases, color and pulsed Doppler ultrasound allow the identification of the regurgitant jet [3–6].

When an AVSD is detected, a complete sequential analysis of the heart is mandatory. Because AVSDs are frequently associated with other cardiac defects. In our study, atrioventricular septal defect was associated with other cardiac anomalies in 34 fetuses (54.8%). Huggon et al. reported 301 fetuses with AVSD. Additional cardiac abnormalities were noted in 146 (48%) of these cases [6]. In the study of Rasiah et al. this finding was observed in 58.58% of the whole series [7]. Friedberg et al. noted that there had been 15 (75%) fetuses with other cardiac anomalies, and the most common cardiac lessions in this study were right ventricular outflow anomalies and aortic arch abnormalities [8].In our series, heterotaxia sydromes or isomerism of the atrial appendages was the most common cardiac lession which was found in 19% of the cases. Double-outlet right ventricle was the second common cardiac lession which was found in 15% of the cases in our study. 

Many of the larger studies reported 13–72% association of AVSD with extracardiac anomalies [6, 9]. In this series significant extracardiac abnormalities and syndromes were identified in 37 of 62 cases, respectively. The range of extracardiac abnormalities was wide and included renal, gastrointestinal, neurological, and skeletal abnormalities. The detection of these associated abnormalities is very important so that when counselling parents an accurate indication of outcome can be given.

Studies indicate 37–58% association of AVSD with chromosomal anomalies, especially trisomy 21 [6, 10]. The fetal karyotype, therefore, should be examined whenever this diagnosis is made. In our series the percentage of fetuses with aneuploidy was 41.17%. Trisomy 21 represented 61.90% of these anomalies. Fesslova et al. reported 82 fetuses with cAVSD. Chromosomal anomalies was noted in 33 (40%) of these cases, and trisomy 21 was found in 28% of the cases [11]. AVSD can also be a part of other syndromes such as Ellis-Van Creveld, VACTRL, CHARGE, Cornelia de Lange, and Goldenhar syndromes [5]. Our patients did not have any stigmata suggesting these syndromes.

Fesslova et al. reported that aneupoidy was a strong predictor for AVSD as an isolated cardiac defect [11]. Huggon et al. reported that fetuses with an isolated defect had a higher incidence of karyotype abnormality than those with complex defect [6]. However in our study there was not any statistical significance between the two groups [*P* = 1.00; RR: 0.86; 95% CI: 0.30–2.50]. We have no explanation for the difference between the studies. Trisomy 18 was less frequent in our series. Triploidy occurred in two cases with associated other intracardiac lesions. Deletion of 46q4 occurred only in one case without any other associated intracardiac lesions.

 Careful examination of the abdominal organs and the systemic veins is essential in the fetus with an AVSD to rule our visceral heterotaxy. This syndome is strongly predictive of a normal karyotype. However in this series, left isomerism and right isomerism were demonstrated in 12 fetuses with AVSD, and abnormal karyotype was found 5 in 12 fetuses (three with trisomy 18 and two with trisomy 13). In all these patients maternal age was above 35, and they were all endogamous. Also there are several reports on the association of cardiosplenic syndrome and chromosomal abnormalities, mainly microdeletion of chromosome 22q11, trisomy 18, and trisomy 13 [6, 9, 12–14]. 

The natural history of complete AVSD is not well understood, since it is unusual for patients with this condition to be treated without surgical intervention. Nevertheless, the addition of an intracardiac shunt at the ventricular level, combined with the atrial level shunt and AV valve insufficiency, makes this a particularly morbid lesion with high mortality if left untreated. A few small series have confirmed this suspicion and suggest that 80% of patients who do not undergosurgery will die by 2 years of age [15, 16].

In the study of Machado et al. which included all fetuses diagnosed with AVSD, only four of the 29 fetuses survived [6]. Huggon et al. observed that their survival rate was 38% by three years of follow up [12]. Friedberg et al. reported that the overall survival rate for fetuses with cAVSD was 40% [8].

The outcome of fetal cAVSD continues to depend on associated lesions, both cardiac, and extracardiac, with a continuing high incidence of pregnancy termination and neonatal death [17]. In our series, a total of 36 parents (58%) opted for termination of pregnancy; 58.4% of those without and 41.6% of those with associated intracardiac lesions. Of 26 cases that continued pregnancy only 22 pregnancies resulted in a live born child. When we exclude termination of pregnancy, the survival rate for fetuses with isolated and complex cAVSD is 69,2% and 30,7%, respectively. 

In conclusion, cAVSD is associated with chromosomal, other cardiac and extracardiac abnormalities. When the diagnosis of cAVSD is made in fetal life, karyotype and a detailed anatomical survey should be offered in every case. The detection of these abnormalities is important in order to give the best indication of the likely outcome when counselling parents.

## Figures and Tables

**Table 1:** Demographic data.

Characteristic	Normal karyotype	Abnormal karyotype	*P*
Mean maternal age (year)*	28.6 ± 4.5 (range 20–38)	29.0 ± 5.2 (range 21–38)	.80
Mean gestational age at diagnosis (week)*	25.9 ± 5.9 (range 12–38)	20.7 ± 2.6 (range 17–26)	.02

*Mean ± standard deviation.

**Table 2:** Indications for performing fetal echocardiograpy.

Indication	*n*	%
Abnormal four-chamber view	16	25.8
Maternal diabetes	6	9.6
Increased fetal NT	11	17.7
Abnormal triple test result	11	17.7
Other abnormalities*	14	22.5
Family history of CHD	4	6.4
Total	62	100

NT: nuchal translucency; CHD: congenital heart disease; *Omphalocele, single umbilical artery, short femur versus.

**Table 3:** Other cardiac anomalies of fetuses with cAVSD.

Cardiac anomaly	*n* : 34	%
Double-outlet right ventricle	4	11.7
Tetralogy of fallot	3	8.8
Double-outlet right ventricle + hypoplasia of the left heart	2	5.8
Dextrocardia	3	8.8
Transposition of the great arteries	3	8.8
Transposition of the great arteries + dextrocardia + pulmonary stenoz	1	2.9
Hypoplasia of the right heart + transposition of the great arteries + ulmonary stenoz stenoz	1	2.9
Right atrial isomerism heart + transposition of the great arteries + pulmonary stenoz stenoz	3	8.8
Left atrial isomerism + double-outletright ventricle	1	2.9
Left atrial isomerism	3	8.8
Right atrial isomerism	4	11.7
Coarctation of the aorta	2	5.8
Hypoplasia of the left heart	2	5.8
Left atrial isomerism + transposition ofthe great arteries + pulmonary stenozstenoz	1	2.9
Double-outlet right ventricle + aorticvalve stenosis	1	2.9

**Table 4:** Extracardiac anomalies associated with cAVSD fetus.

Anomalies		*n* : 62*	Total %
CNS	Arnold-chiari malformations	2	(22,5%)
	Holoprosencephaly	1	
	Mild ventriculomegaly	1	
	Agenesis of the corpus callosum	1	
	Strawberry-shaped head	1	
	Choroid plexus cysts	5	
	Dandy-walker syndrome	1	
	Exencephaly	1	
	Iniencephaly	2	
	Total	14	
GIS	Omphalocele	5	(17,7%)
	Duodenal atresia	2	
	Hyperechogenic bowel	4	
	Total	11	
AE	Club foot	2	(17,7%)
	Polydactyly	1	
	Short femur	5	
	Clinodactyly	1	
	Rocker-bottom foot	1	
	Short humerus	1	
	Total	11	
FA	Cleft lip and/or cleft palate	1	(9,6%)
	Microphthalmia	1	
	Flat facial profile	2	
	Small nasal bone	2	
	Total	6	
GUS	Bilateral renal pelvic dilatation	3	(6,4%)
	Multicystic dysplastic kidney	1	
	Total	4	
Other anomalies	Nuchal fold	4	
	Fetal hydrops	3	
	Single umbilical artery	8	
	Ivemark syndrome	11	

CNS; central nervous system, GIS; gastrointestinal system, AE; anomalies of the extremites, FA; facial anomalies, GUS; genitourinary sytem.

*A case can have more than one anomaly and hence the number of anomalies will exceed the total number.

**Table 5:** Comparison of isolated and complex cAVSD cases.

Parameters	Isolated cAVSD	Complex cAVSD	*P* value	Relative risk
	(*n* : 28)	(*n* : 34)		(95% CI)
Age (year, mean ± SD)	28.9 ± 4.7	28.6 ± 4.9	0.79	1.23 (−2.1, 2.7)
Gestational age (weeks, mean ± SD)	23.9 ± 6.5	23.5 ± 4.5	0.76	1.41 (−2.3, 3.2)
Extracardiac anomalies (%)	10 (35.7%)	11 (32.4%)	0.49	1.16 (0.4, 4.4)
Choromosome abnormality (%)	9 (42.9%)	12 (51.1%)	1.0	0,89 (0.3, 2.5)
Terminations (%)	15 (41,7%)	21 (58,3%)	0.6	0.71 (0.2, 1.9)
In utero mort fetus (%)	0	4 (11,8%)	0.12	0.51 (0.4, 0.6)
Live born (%)	13 (% 59.1)	9 (40.9%)	0.11	0.41 (0.14, 1.2)
Overall survival to date (%)	9 (69.2%)	4 (30.8%)	0.28	0.28 (0.07, 1.04)

**Table 6:** Cases with intrauterine demise.

Gestational age at intrauterine demise (weeks)	Type of cAVSD	Additional cardiac defect Karyotype	Additional noncardiac defect	Karyotype
25	Complex	Atrioventricular block and severe insufficiency of the atrioventricular valve	Bilateral renal pelvic dilatation and fetal hydrops	Normal
27	Complex	Double outlet right ventricle and pulmonary stenosis	Dodenal Atresia, Cleft Lip, and/or Cleft Palate and Fetal Hydrops	Normal
30	Complex	Double outlet right ventricle and left atrial isomerism	None	Normal
26	Complex	Double outlet right ventricle and left atrial isomerism	None	Normal
